# Personality, self-efficacy and coping in Romanian prison inmates: a moderated mediation model

**DOI:** 10.3389/fpsyg.2025.1544733

**Published:** 2025-10-02

**Authors:** Cornelia Rada, Flavia-Elena Ciurbea, Robert-Andrei Lunga, Andreea-Cătălina Forțu

**Affiliations:** ^1^Biomedical Department, Francisc I. Rainer Institute of Anthropology, Romanian Academy, Bucharest, Romania; ^2^Penitentiary Police National School “Constantin Brâncoveanu” Târgu Ocna, Bacău, Romania; ^3^Department of Psychiatry, ‘Prof. Dr. Alexandru Obregia’ Clinical Hospital of Psychiatry, Bucharest, Romania; ^4^Personnel Psychology Service, National Administration of Penitentiaries, Bucharest, Romania

**Keywords:** personality, self-efficacy, coping, crime, inmate, prison, detention

## Abstract

**Introduction:**

This study investigates personality traits, coping mechanisms, and the mediating function of self-efficacy in the relationship between these factors in individuals incarcerated for criminal offenses.

**Methods:**

The Freiburg Personality Inventory, COPE Inventory, and Self-Efficacy Scale were used to assess the responses of 857 inmates. The moderating influence of age, sex, and number of years of incarceration was examined using a mediation model.

**Results:**

The participants reported high scores on the aggressiveness, strain, somatic complaints, health concern, and emotionality scales (Cronbach α > 0.7; their composite score *p* < 0.001). Self-efficacy and the negative indirect effects of personality on coping mechanisms (*p* < 0.05) highlighted the indirect negative effects of mediation on the personality-coping relationship. Age reduced self-efficacy and additional coping mechanisms, and a high emotionality score negatively impacted self-efficacy (*p* < 0.001). When the incarceration duration was less than five years and the personality score increased, coping mechanisms were more prominent.

**Discussion:**

Owing to their health concerns, feelings of being overworked, psycho-vegetative lability, and resistance to the desire to express themselves verbally and physically, detainees require evaluation and intervention. When individuals gain confidence in their own skills to overcome the period of detention and rectify themselves behaviorally, emotional lability is reduced and a suitable context for educational and psychotherapy intervention is created. The distinctiveness of personality, coping strategies, self-efficacy, sex, age, and years of incarceration, should all be considered in correctional programs and activities.

## 1 Introduction

Individuals enter the penitentiary with a personality profile, and some of them, if they end up in prison, probably have an antisocial personality disorder. At the same time, the prison environment and detention impact prisoners’ personalities.

The act of breaking the law is punished by imprisonment, and the offender is expected to realize that he has done something wrong, rectify himself, and not commit wrongdoing again. However, the process of education and re-education is not simple, and sometimes renders no results.

Educational programs in prisons aim to develop inmates’ independence and personality, as well as healthy coping mechanisms. However, in the case of repeat offenders, the situation is even more complex. They sometimes become involved in activities outside of obligation or refuse to participate altogether; they are likely to have not attended such programs in the past, consider these activities pointless, become depressed, and even end up in prison again (Meyer et al., 2014; Mutz and Müller, 2023).

Thus, the following question arises: What are the factors that influence adjustment and remediation, which lead to a decrease in recidivism?

The behavior of detainees and their adjustment to prison are not only influenced by the conditions within the facility but are also affected by a series of factors preceding incarceration, such as criminal history, attitudes towards violence, mental health issues, traumatic experiences, abuse, and marginalization (DeLisi et al., 2011). A potentially traumatic history, low levels of external support (family, friends), defeatism, denial of reality, and excessive use of avoidance or direct confrontation diminish the ability to adapt (Leban et al., 2016).

Langenhoven et al. (2024) found that the depressive mood of prisoners is influenced by age, perception of social support, coping mechanisms, and psychological and behavioral adaptation to the conditions and rules of the prison environment.

Longer time spent in prison, inhumane treatment, negative experiences in detention, and a negative perception of procedural justice, certain negative experiences, such as lack of social support, violence, incarceration conditions, and absence of constructive activities, are significant factors of maladaptation in prison (Butler et al., 2021; Butler, 2022).

A criminal life history (e.g., frequent criminal activities, criminal environments, negative social values and norms, drug abuse, lack of a stable life) and psychopathic traits decrease adjustment in prison (Pinheiro et al., 2021).

Adaptation to incarceration is not a single event but a continuous process that evolves throughout the detention period. The longitudinal study conducted by Kovács et al. (2019), highlighted that as the incarceration period progresses, inmates can develop coping strategies and internal resources that facilitate more effective adaptation. This adaptation process is influenced by several factors, including the individual’s personality traits, contact with the outside world through visits, and the qualitative level of economic and educational conditions within the prison.

Some authors appreciate the detrimental effect of imprisonment on mental and physical health, both during the actual time served and afterwards. It also appears that imprisonment does not rehabilitate individuals to the extent expected (Cunha et al., 2023).

Studies have shown that most incarcerated people have common mental disorders (Fentahun et al., 2023). Most studies have found a deterioration of physical and mental health in imprisoned people (Mutz and Müller, 2023). Although experiencing imprisonment may lead to health problems before, during, and after actual incarceration, some studies have found that health did not deteriorate because of imprisonment, but did not show any improvement either (Dirkzwager et al., 2021). However, some research has shown an improvement in health status in those who prior to incarceration were in disadvantaged situations regarding food, shelter, and health care, or who had attended different support programs, sports, occupational therapy, and psychotherapy during incarceration (Wallace and Wang, 2020).

According to 2020 statistics, the expenditure per day per prisoner in Romania amounted to 43€, 21€ lower than the average in European countries (Asociația Voci pentru Democraţie şi Justiţie, 2020). Additionally the turnover rate of prisoners in Romania was 11% lower than in Europe, which indicates relatively long periods of detention and can be interpreted as an alarm signal regarding the risk of prison overcrowding (Aebi and Tiago, 2021).

According to the annual activity report for the year 2022 of the National Penitentiary Administration, among the chronic conditions that prisoners were registered with, the highest proportion was for mental disorders, followed by cardiovascular diseases (Administrația Națională a Penitenciarelor, 2023).

Based on individuals’ beliefs that they possess effective skills to perform an action successfully, self-efficacy becomes a key trait that strongly influences the way individuals engage in various activities (Bandura, 1977).

Freire et al. (2020) found that students’ self-efficacy could be positively influenced by more flexible use of coping strategies. In his study of 589 prison inmates in New Mexico, Ravenscroft (2022) found that those who did not experience a sense of powerlessness in problem solving scored higher on perceived crime-free coping. Some studies have indicated that higher self-efficacy scores have positive effects on decreasing recidivism (Woldgabreal et al., 2016; Cuevas et al., 2017). However, other studies have found a correlation between self-efficacy and the level of criminality in the workplace (Muthuswamy and Varshika, 2023) and that self-efficacy may be a significant predictor of Machiavellianism (Zaman and Qayyum, 2020), while the two factors may have the potential to hinder the social reintegration process.

Being in prison is a stressful experience; therefore, coping mechanisms may play an important role in adapting to this environment to limit distress and self-harm. Luke et al. (2021) found that avoidance coping mechanisms were associated with distress and that seeking social support was associated with lower levels of distress in older adult prisoners. In their study on prisoners in Poland, Leszko et al. (2019) found that conscientiousness was positively associated with task-oriented coping and negatively associated with emotion-oriented coping, and neuroticism was negatively associated with task-oriented coping.

The results of the variability in the coping mechanisms used were also not unanimous. It seems better to view personality, self-efficacy, and coping from the perspective of mutual mediation. By understanding personality traits, psychological interventions can become more effective; consequently, prisoners’ coping skills in relation to stress will improve. Consequently, this study provides further information and clarifies the role of self-efficacy.

Therefore, the following research questions were formulated concerning jail inmates: Is there specificity in personality? What role does self-efficacy play in the adaptation process and what variables may be involved in achieving better mental health and resilience?

The results of this study will provide information that can constitute the basis for the development and implementation of specific measures to improve mental health and coping skills in prisoners and to help them achieve good reintegration into society as well as a decrease in recidivism.

This study is in line with Goal 16 of the sustainable development goals proposed at the 2030 Agenda for Sustainable Development of the United Nations (2023): Promote peaceful and inclusive societies for sustainable development; provide access to justice for all; and build effective, accountable, and inclusive institutions at all levels.

## 2 Materials and methods

### 2.1 Procedure

After submitting the project documentation to the National Administration of Penitentiaries (ANP) in Romania and receiving an *agreement in principle*, a request for access was sent to each prison. Out of the 43 prisons in Romania, seven responded positively to the request for the study, namely Aiud, Arad, Craiova, Baia Mare, Codlea, Târgu Mureș and Târgu Jiu.

Between July 2021 and August 2022, data were collected from 857 prisoners in agreement with the ANP. The selection criteria for the respondents were as follows: aged over 18 years, their penalties must be execution sentences, and possession of writing and reading skills. Several steps were necessary to gain access to the prison environment. These steps included sending a request for acceptance to the director of the ANP regarding collaboration and permission to enter Romanian prisons together with the research project plan, and details of the research objectives, including information on the number of participants required. This stage was finalized with the *agreement in principle of* the National Penitentiary Administration. The next stage consisted of sending a request for access to each prison management, with information on the objectives of the research, the required number of participants and the period during which the research was intended to be carried out.

Within each penitentiary, work was conducted by detention sectors. The regimes for the execution of custodial sentences are maximum security, closed regime, semi-open regime, and open regime. The schedule for administering the test battery was outlined according to the program of each sector. Participants were invited, one per room in each section, to complete the survey. They were informed about the possibility of withdrawing from the research at any time without any repercussions.

The Ethics Committee of the “Constantin Rădulescu-Motru” Institute of Philosophy and Psychology, Romanian Academy, Bucharest, approved the research in 03.12.2019 (certificate 73/10-07-2020). This study followed the principles of the Declaration of Helsinki to grant respect for human rights. The respondents provided written consent for participation, and that the European and national data processing standards were respected in each stage of the research.

The research objectives were as follows:

1. To create a personality profile of those convicted and imprisoned.
2. To identify how personality and coping mechanisms relate to each other.
3. To highlight the mediating role of self-efficacy in the relationship between personality and coping mechanisms in prisoners.

The Self-Efficacy Scale Questionnaire (SES) developed by Schwarzer and Jerusalem (1995), the Coping Orientation to Problems Experienced (COPE Inventory) (Carver et al., 1989), and the Freiburg Personality Inventory (FPI-R) (Fahrenberg et al., 2001) were used to achieve the objectives.

This study uses the concept of mediation as a process in which one variable transmits an effect to another through one or more mediating variables. It also refers to moderation, that is, how a particular variable changes the direction or strength of the association between the two variables. Identifying the moderator variables clarifies the relationship between two variables. The moderator is not part of the causal process in question, but interacts with the relationship between the two variables in such a way that, depending on the values of the moderator, their relationship is stronger, weaker, or opposite in direction. Conceptually, we examined the conditional indirect effect by quantifying the indirect effect at different moderator values. Depending on the value of the moderator, the indirect effect may be stronger, weaker, or opposite in sign. It is also taken into account that a moderator can act on any relation that is part of a proposed mediation model.

### 2.2 Tools, consistency analysis

Self-efficacy refers to an individual’s belief in their ability to perform the behaviors necessary to achieve specific performance. The Self-Efficacy Scale (SES) developed by Schwarzer and Jerusalem (1995) and validated in Romania by Vasiliu et al. (2015) was used to measure self-efficacy.

This scale contains 10 items rated on a four-point Likert scale ranging from one to four. The Romanian version of the SES allows us to obtain a global indicator of self-efficacy in a 5-level score standard.

The internal consistency of the 10-item SES questionnaire was good, with a Cronbach’s α coefficient of 0.886. The confirmatory factor analysis of the one-factor model indicated a good fit for the sample data: CFI = 0.997, TLI = 0.995, RMSEA = 0.048, and RMSEA *p* = 0.596.

The self-efficacy score was calculated as the sum of the scores of the 10 items, with values ranging between 10 and 40. The mean self-efficacy score was M = 31.17 (SD = 6.55).

#### 2.2.1 Coping inventory

Coping strategies were assessed using the COPE Inventory (Carver et al., 1989), which comprises 60 items, validated in Romania in 2013 by Crașovan and Sava. The instrument initially assessed 15 coping strategies: use of humor, positive interpretation and personal development, mental detachment, focus on expressing emotions, use of instrumental social support, active problem approach, denial, religious approach, behavioral detachment, refraining, use of emotional support from the community, substance use, acceptance, suppression of competing activities, and planning. Responses using a Likert scale ranging from 1 to 4.

The internal consistency of the 15 coping mechanisms ranged between α = 0.467 and α = 0.849 and was, with four exceptions, not acceptable, as the Cronbach’s α coefficient was below 0.7. For this reason, as proposed by Crașovan and Sava (2013), a more compact model was used with only four coping mechanisms: emotion-focused coping, problem-focused coping, social support-based coping and avoidance coping.

For the four new mechanisms, 48 items (12 mechanisms) from the original COPE Inventory were used (compact models). In this reduced model, internal consistency improved. For each mechanism, Cronbach’s α coefficient had values above 0.7. The CFA analysis of the four-mechanism model showed modest performances: CFI = 0.857, RMSEA = 0.094, TLI = 0.839, but comparable to those obtained by Crașovan and Sava and acceptable for the purpose of this research. Each mechanism was independently analyzed using CFA. The indicators of model quality (goodness of fit) had satisfactory values, and raw scores were calculated for each mechanism as the sum of the corresponding items.

#### 2.2.2 Personality

The Freiburg Personality Inventory (FPI-R), a multi-item scale developed by Fahrenberg et al. (2001), was used to assess personality dimensions according to the adult multiphasic model. The FPI-R was adapted and validated for Romanians in 2015. The national normative reference sample for Romania included 2,400 participants (1,200 women and 1,200 men). For the 12 FPI-R personality measurement scale, life satisfaction (LEB), social orientation (SOZ), achievement orientation (LEI), inhibitedness (GEH), excitability (ERR), aggressiveness (AGGR), strain (BEAN), somatic complains (KORP), health concern (GES), frankness (OFF), extraversion (E), and emotionality (N) raw scores were calculated using the sum of the scores (0 = False or 1 = True), with groups of 12 or 14 items, from the 138 items indicated in the Freiburger test methodology.

The internal consistency analysis indicated that the Cronbach’s alpha coefficient has an acceptable α value greater than 0.7 for only 5 scales: the AGGR with 12 items (α = 0.775), BEAN with 12 items (α = 0.777), KORP with 12 items (α = 0.780), GES with 12 items (α = 0.708), and N with 14 items (α = 0.805). These scales and their corresponding scores were retained for further analysis.

Confirmatory factor analysis of the five personality traits indicated a single-factor model as appropriate for the data: CFI = 0.996, TLI = 0.992, RMSEA = 0.048, and RMSEAp = 0.486. All correlation coefficients between the personality factor and the scale scores (factor loadings) were statistically significant with a maximum value of λ = 0.974 for the N score (emotionality) indicating the variance explained by the variable through the associated factor. Values above 0.8 were observed for BEAN (0.864) and KORP (0.805), while values below 0.7 were observed for AGGR and GES.

The internal consistency for the unifactorial model with the five items (AGGR, BEAN, KORP, GES, N) was good, α = 0.821. A component score was calculated as the sum of the scores for each scale, which correlates and reliably estimates the personality factor score [Pearson’s r(845) = 0.950, *p* < 0.001]. High component scores (sum of the scores on each scale) were mainly associated with a high score on the personality trait emotionality. Neuroticism (λ = 0.974). This score was used for subsequent analyses.

#### 2.2.3 Moderated mediation model

**Figure 1:**
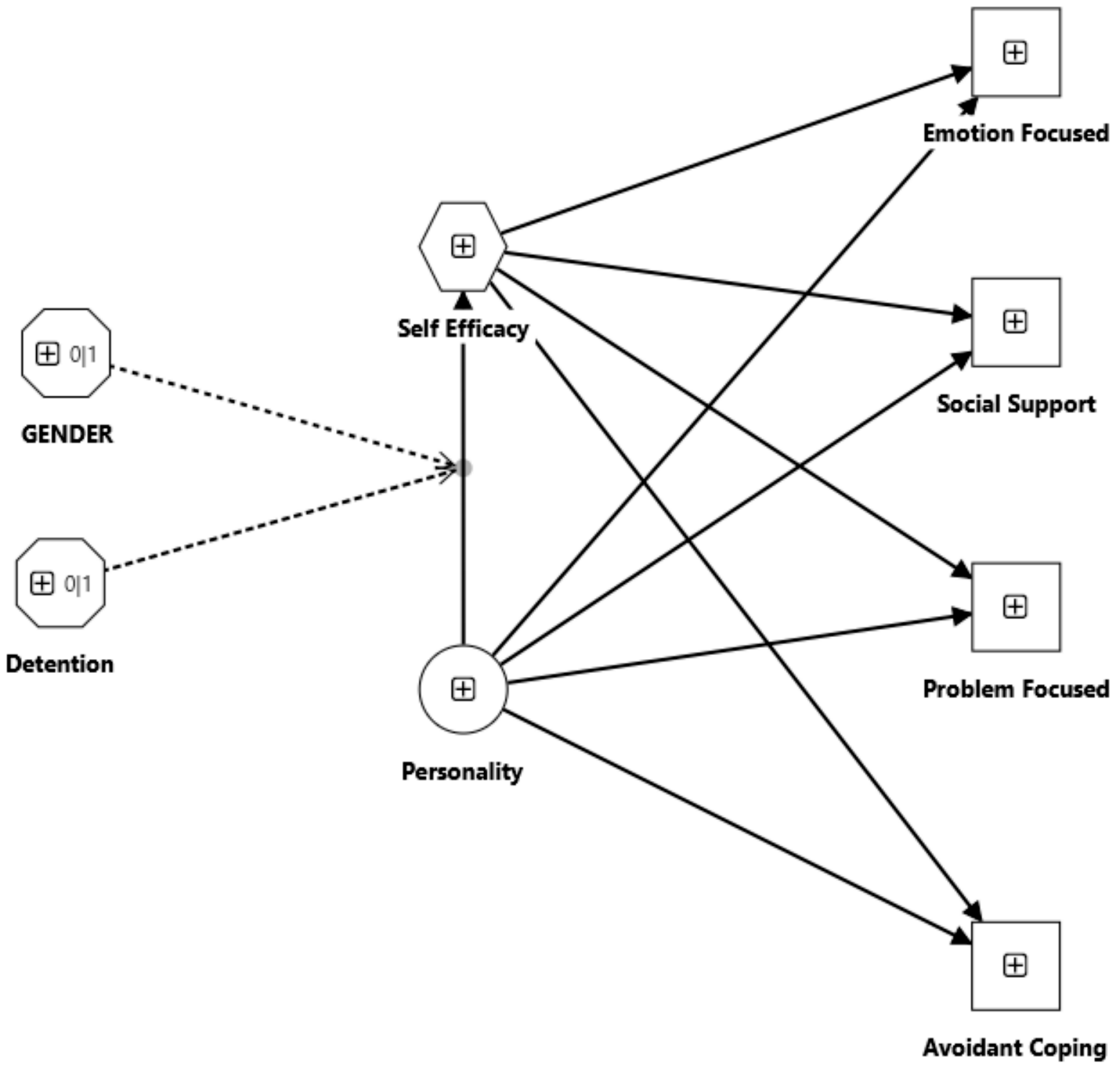
Conceptual moderated mediation model.

This study aimed to highlight the mediating role of self-efficacy in the relationship between prisoners’ personality and their coping mechanisms. A conceptual model developed using SmartPLS 4 (Ringle et al., 2022) is schematically presented in Figure 1. The model variables were previously calculated as raw scores for personality (independent variable), self-efficacy (mediator), and coping mechanisms (dependent variable). Two binary moderators were also introduced into the model in the next step: sex (male, female) and period of incarceration (under 5 years, over 5 years), which, by hypothesis, affect the relationship between personality and self-efficacy.

The scores of the variables in the model are presented in Table 1.

**Table 1:** Mean scores by sex and years of detention.

Variables	Sex		Detention	
Scores	Men	Women	<=5 years	>5 years
Self-efficacy	31	33	32	31
Personality	28	22	27	29
Emotion focused	34.40	36.21	34.72	34.49
Problem focused	33.57	35.30	33.23	34.15
Social support	28.97	32.81	29.17	29.54
Avoidant coping	23.45	25.03	23.27	23.89

AGGR scale with 12 items (α = 0.775), BEAN scale with 12 items (α = 0.777), KORP scale with 12 items (α = 0.780), GES scale with 12 items (α = 0.708), and N scale with 14 items (α = 0.805).

## 3 Results

For all four coping mechanisms analyzed in relation to the personality scores, the simple mediation model without moderators revealed significant indirect negative mediation effects in the personality-coping relationship through self-efficacy (*p* < 0.05).

**Figure 2:**
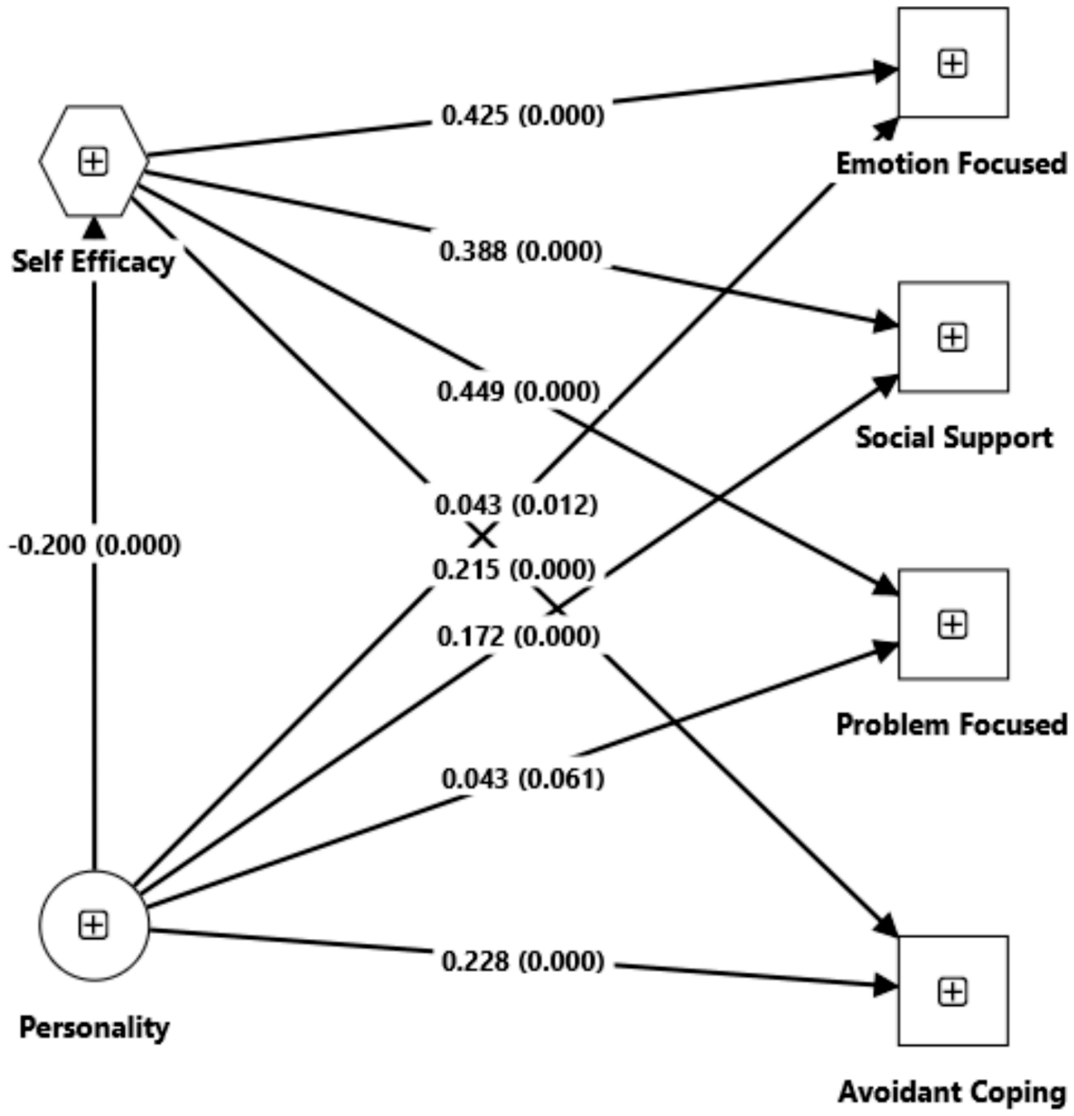
Simple mediation SEM.

The direct effects of personality on coping mechanisms (*p* < 0.05) were also significant in the simple mediation model without moderators. The direct effects were positive, indicating that an increase in personality scores increased the coping scores (without the self-efficacy mediator). In contrast, negative indirect effects (when self-efficacy was involved) indicated the attenuation of coping scores when personality scores increased. Simple mediation SEM graphical presentation is shown in Figure 2.

The estimation of indirect effects and the confidence intervals obtained through bootstrapping with JASP version 0.18.1 (JASP Team, 2023) are presented in Table 2.

**Table 2:** Indirect simple mediation effects.

Coping Mechanism	Estimate	Std. Error	*z*-value	*p*	95% CI	
					Lower	Upper
Emotion focused	−0.086	0.010	−8.608	< 0.001	−0.107	−0.065
Problem focused	−0.093	0.012	−8.029	< 0.001	−0.119	−0.070
Social support	−0.077	0.010	−7.741	< 0.001	−0.101	−0.057
Avoidant coping	−0.046	0.007	−6.117	< 0.001	−0.064	−0.031

Delta method standard errors, bias-corrected percentile bootstrap confidence intervals, ML estimator.

The total effects (c) of personality on coping mechanisms mediated by self-efficacy, measured as the sum of the direct and indirect effects, were also significant (zero was not included in the confidence interval), as follows: emotion-focused coping mechanism c = − 0.042, 95%CI [− 0.079; − 0.004], problem-focused coping c = − 0.047, 95%CI [−0.089, − 0.004], social support-based coping, c = 0.094, 95%CI [0.062, 0.136]; and avoidance-based coping mechanism, c = 0.185, 95%CI [0.153, 0.221]. Negative total effects indicated a decrease in coping scores when personality scores increased, and positive total effects indicated an increase in coping scores when personality scores increased. These scores confirm that self-efficacy mediates the relationship between personality and coping.

For the relationship between the self-efficacy mediator and each coping mechanism, the regression coefficient (b) was positive and significantly different from zero in all cases. Refer to Figure 2.

Specific to the sample of people in detention, personality, where a high score indicates emotional instability (neuroticism), negatively influences self-efficacy, with a regression coefficient a = −0.200, 95%CI [−0.2379, −0.154], which is the cause of the negative indirect effect (product of coefficients a and b) obtained by self-efficacy mediation (Hayes, 2013).

The results obtained in this step support the hypothesis that the personality effect is transmitted to coping mechanisms, mediated by self-efficacy, and that individuals’ beliefs in their ability to act in the ways are necessary to achieve specific goals. In the sample analyzed, this belief was attenuated when personality was strongly neurotic, and consequently, coping mechanisms were attenuated. The negative indirect effect diminishes the positive direct effect (c’) of personality on coping.

In the proposed moderated mediation model, the two binary moderators–sex (male, female) and period of incarceration (under 5 years, over 5 years)–only affect the relationship between personality and self-efficacy (coefficient a).

Moderated mediation analysis, performed using the PROCESS macro (Hayes, 2022, Version 4.3) in SPSS (IBM Corp, 2020), showed that for coping mechanisms, the partial moderated mediation indices (Hayes, 2018) for both moderators (sex and imprisonment) were significantly different from zero, which confirms the hypothesis that the indirect effect is related to the moderators. For female prisoners with sentences over 5 years, this mediation was not identified.

The indirect effects of personality on self-efficacy-mediated coping mechanisms conditioned by moderators are presented in Table 3.

**Table 3:** Conditional indirect effects of personality on coping mechanisms.

Moderators		Coping mechanisms			
Sex	Detention	Emotion	Problem	Social	Avoidance
Men	< 5 years	−0.0560	−0.0608	−0.0501	−0.0298
Men	> 5 years	−0.1291	−0.1402	−0.1156	−0.0688
Women	< 5 years	0.1112	0.1208	0.0996	0.0592
Women	> 5 years	0.0381^*^	0.0413^*^	0.0341^*^	0.0203^*^

* Not significant because zero is included in the bootstrap 95% confidence interval.

In contrast to the simple mediation model, in the moderated model, the indirect effect was positive for female prisoners, and consequently, coping mechanisms were enhanced when the personality score increased. The effect decreased in persons sentenced to more than 5 years to small values that were not significantly different from zero.

In women, when the period of imprisonment was less than 5 years, all four coping mechanisms were enhanced. In the case of men, the indirect effect was negative and larger in absolute value if the sentence was more than 5 years, causing an attenuation of coping mechanisms when the personality score increased. In men, when the period of conviction was longer, coping mechanisms were attenuated.

When the binary mediator age group (under 40 years and over 40 years) was introduced separately into the simple mediation model, a significant negative indirect conditional effect was found, which was stronger in the group aged over 40 years. For the same personality score, age decreased self-efficacy and coping mechanisms.

## 4 Discussion

### 4.1 Dimensions of personality in prisoners

The emotionality scale of the FPI-R was constructed by the authors, similar to the personality trait neuroticism, described by Eysenck and Eysenck (1969) and by McCrae and Costa (1999) in the Big Five Factor Theory. High scores on the personality dimension emotionality characterized this sample of inmates; that is, these individuals were neurotic, emotionally unstable, and anxious.

Given how this scale is described when it has high scores, on the one hand, easily excitable and easily provoked, and, on the other hand, asthenic and lacking energy, it is necessary to consider interventions for emotional rebalancing. The authors of the questionnaire found that high emotionality scores correlated with high strain, somatic complaints, and health concern scores. This was also confirmed in our sample, which had high scores on strain (BEAN), somatic complains (KORP), and health concern (GES) scales. All five dimensions were characteristic of the sample; however, emotionality was the most common.

This combination of high emotionality and aggression scores needs to be considered in intervention programs because studies have shown associations between negative emotionality and physical aggression (Garofalo and Velotti, 2017). Emotional life in prisons, even when routine, raises a number of issues, such as inmates’ anger at the deteriorating conditions of detention, the way they are treated by guards, and the fear of being attacked by other prisoners.

A prison is a highly structured, rigid, socially isolated, and hostile environment, where individuals are at risk of attack from other prisoners (individually or in gangs), which can destroy personality. Such a traumatic environment during imprisonment can lead to post-traumatic stress disorder (PTSD), especially in the case of homicide offenders and those with serious mental illnesses at the time of the crime (Belet et al., 2020).

High scores on the strain, somatic complaints, health concerns, emotionality, and aggressiveness scales obtained from this sample of prisoners may be owing to symptoms of PTSD. These require intervention not only during detention, but also after release.

Emotions with negative valence, such as fear, sadness, and anger, should be treated separately, as anger may be associated with aggression but is less likely to be associated with fear and sadness. Emotional distress is implicated in violence, suicide, and anxiety, which can lead to anger (Yasmin et al., 2022; Macassa et al., 2023).

Considering how self-efficacy can be a mediating factor between coping and personality, interventions to cultivate the emotions of hope and enthusiasm would help inmates cope and think in a different way, so that they do not commit more crimes.

Prisoners in this sample reported feeling tense and strained. It should be noted that the strain scale captures not specifically assess actual overstrain, but the subjective experience of overstrain, such as defeatism and the habit of complaining. The somatic complaints scale shows that participants have asthenic and pessimistic behavior and a psycho-vegetative lability that they usually verbalize.

Similar to other studies (Ifeagwazi et al., 2020), the present research emphasizes the need to assess emotion regulation and awareness of affective dispositions, as they are common to somatic symptoms.

Future studies are needed to identify the extent to which the prison population suffers from somatoform disorders, considering that they are comorbid with personality disorders and substance abuse.

High scores on health concerns indicate that prisoners worry about their own health, possible accidents, low robustness, and anxiety. This needs to be considered by prison staff for remediation, as studies have shown that those with physical and mental health problems are at risk of engaging in inappropriate behaviors in prison (Semenza and Grosholz, 2019; Canada et al., 2022).

Agnew (1992) found that individuals experience three types of strain: (a) failure or anticipation of failure to achieve goals rated as positive, (b) actual or anticipated elimination of stimuli rated as positive, and (c) actual or anticipated presence of stimuli rated as negative. If individuals lack adequate coping mechanisms, the anger and frustration generated by these stressors can lead to criminal or delinquent behavior. Mental health is essential in the context of liberty deprivation, as it allows for the correct perception of stimuli and a state of well-being with favorable adaptive effects, both in prison and after release. Good mental health also reduces recidivism (Cunha et al., 2023).

It is also necessary to consider the high score on the aggressiveness personality dimension, which denotes a propensity toward aggressive, reactive, and sometimes spontaneous behaviors, indicating low self-control. As described by the authors of the Freiburg Personality Inventory Aggressiveness Scale, it appears that the prisoners in this sample have a tendency to violate social norms. They must learn not to use force or frontal confrontations to respect their rights, and that the response to provocation is neither verbal nor physical. The prison environment, more than other environments, also entails the risk of assaults and violations of rights, to which they must learn to respond by means other than force. Learning about self-control is essential, especially for violent offenders.

### 4.2 Self-efficacy as a mediator in the relationship between personality and coping mechanisms in prisoners. Moderating factors

Self-efficacy is one’s belief that one’s own actions can be or are responsible for the success of a particular activity. The belief that one can effectively cope, especially in a deprived environment, is particularly important. Bahr et al. (2010) found that low self-efficacy was associated with a negative attitude toward authority and implicitly with a refusal to perform detention-specific tasks.

However, experiencing negative feelings while in prison leads offenders to believe that perfecting dysfunctional behavior will help them achieve the well-being they desire. As a result, while serving their sentence, they may seek to become more effective in their criminal behavior (Cornish and Clarke, 2014).

A simple mediation model (without moderators) indicated that an increase in the personality dimensions of aggressiveness, strain, somatic complaints, health concerns, and emotionality led to an increase in coping scores. Consequently, these negative personality dimensions cause prisoners to seek coping mechanisms intensively to cope with the activation of these dimensions in the custodial environment. In contrast, when self-efficacy was implicated, it personality scores on the personality dimensions increased, attenuating coping scores. These findings confirm that self-efficacy is the mediating factor between personality and coping mechanisms.

The involvement of self-efficacy in the relationship between personality and coping mechanisms is a central idea of the present study because acting on this mediator could have positive effects on negative personality dimensions and coping mechanisms in a deprived environment. This idea is supported by Skowroński and Talik (2023), who found that in a sample of almost 400 men incarcerated in correctional institutions, self-efficacy was positively correlated with quality of life and that ego-resiliency increased.

Scores on personality traits such as aggressiveness, strain, somatic complaints, health concerns, and emotionality can be decreased by increasing individuals’ sense of self-efficacy. In addition, it is necessary to direct self-efficacy toward prosocial behaviors by involving prisoners in re-education activities. Prisoners’ relationships with prison staff play a major role in preventing the perpetuation of criminal self-efficacy (Ung and Lloyd, 2024).

Another aspect highlighted in the present study was that decreasing scores on personality dimensions have different effects on coping. Thus, decreasing scores on personality will lead to increasing scores on emotion-focused and problem-focused coping mechanisms (which had a negative coefficient), and decreasing scores on social support and avoidance coping mechanisms (which had a positive coefficient).

Although problem- and emotion-focused coping have distinct proximal goals, they can facilitate each other, and it is not wise to categorically state that one is good and the other bad. Instead, interventions are required to improve their effectiveness. For example, effective emotion-focused coping decreases negative distress, making it possible to analyze the problem calmly and provide a framework for problem-focused coping (Stanisławski, 2019).

Concrete interventions in re-education programs require a reduction in adaptation through moral/hedonic disengagement. One way is to learn about self-compassion and empathy. Yang et al. (2020) found that self-compassion decreases the tendency toward unethical behavior and moral disengagement. In addition, in switching to rule-following, moral decisions may decrease the risk of undesirable behaviors, as found by Achar and Lee (2022).

Further research is required to explain why lower scores on the AGGR, BEAN, KORP, GES, and N scales activate different coping mechanisms.

The mutual influences between these personality dimensions and self-efficacy are complex because in this study, high emotional scores decreased self-efficacy. Similar results were also obtained in the general population (Doménech et al., 2024; Ramos-Cejudo et al., 2024). High emotionality is characteristic of the prisoner sample.

Self-efficacy and emotional instability potentiate one another. Therefore, acting in the sense of increasing self-confidence in their own abilities to overcome the detention period with all its challenges could help decrease their lability to obtain a more relaxed, optimistic framework, which would make it easier to intervene therapeutically, especially as they are excitable and easily provoked.

In the moderated mediation model with sex and period of imprisonment as moderators, it was found that in women with a period of imprisonment of less than 5 years, all coping mechanisms were emphasized. In men with longer detention periods, coping mechanisms were attenuated. When introducing the age group mediator in the simple mediation model (under 40 years and over 40 years), it was found that at the same personality score, self-efficacy decreased with age, and consequently, coping mechanisms. Additionally, Meyers et al. (2024) found that changes in coping during the first 6 and 12 months of incarceration were associated with higher levels of adverse mental health symptoms. Changes in emotional and problem-focused coping were not associated with mental health symptoms. These results indicate that the design of prison systems should consider the period of incarceration, sex, and age of the prisoner because responses to stressful events, as well as self-efficacy, may vary according to these factors.

This study has some limitations. First, data for this study were self-reported. Second, it was unbalanced in its gender structure. Third, it was not representative of the entire prison population at the time of collection. However, the large sample size, variety of instruments, especially the complexity of the Freiburg Personality Inventory and statistical methods make this study a fairly consistent framework for designing ameliorative and preventive intervention frameworks.

Responses to alcohol and drug use obtained through the COPE questionnaire may have low accuracy in the prison context, where such behaviors are explicitly prohibited. This situation may lead to an underestimation of the prevalence and intensity of these behaviors, since participants may be motivated to provide inaccurate answers to avoid negative repercussions or to maintain a favorable social image (prestige reaction). To achieve a relative overcoming of this limit, the investigator insisted on the following idea: how the respondent would proceed if he had access.

It has been considered that these items are relatively useful because, although legislation prohibits the consumption of alcohol and drugs in penitentiary, the reality shows that some inmates continue to find clandestine ways to obtain them from outsiders or produce them themselves using improvised methods (Norman, 2023).

The desire for a temporary escape from the harsh reality of detention, the reduction of emotional or physical pain, or even to demonstrate their status within the prison hierarchy leads inmates to consume substances and seek illegal ways to obtain them. In the future, more attention should be paid to this issue through the use of interviews and narratives, especially since some inmates enter prison with substance use disorders (Novarizal and Hasan, 2025).

## 5 Conclusion

This study confirmed the hypothesis that personality effects are transmitted to coping mechanisms mediated by self-efficacy. Belief in individuals’ ability to act in ways necessary to achieve specific goals is attenuated when the personality is highly neurotic; as a result, coping mechanisms are also attenuated.

The personality profile of the inmates in the Romanian sample showed neuroticism, emotional instability, anxiety, excitability, and a lack of energy (high scores on the emotionality scale). They felt overstrained, had psycho-vegetative lability, and were worried about their health (high scores on the strain, somatic complaints, and health concern scales). Simultaneously, a tendency toward verbal and physical aggression, threat, and revenge (high scores on the aggressiveness scale) was observed. Some of these symptoms may indicate PTSD, which requires assessment and intervention both during and after detention.

Special attention should be paid to the aggressive personality dimension, which indicates prisoners’ tendencies to violate social norms. Re-education programs should be conducted for prisoners to learn about respect for rights and the difference between acting and reacting.

The central idea of this study was the involvement of self-efficacy in the relationship between personality and coping mechanisms. Increased feelings of self-efficacy would lead to lower scores on personality dimensions that are counterproductive, and consequently, impact coping during detention. In addition, it is necessary to direct self-efficacy toward prosocial behaviors by involving prisoners in re-education activities. Cooperative relationships between prison staff and prisoners can play an important role.

Another important conclusion is that self-efficacy decreases emotionality scores, which shows the complexity of mutual influences between them and the fact that they potentiate each other. Consequently, acting to increase self-confidence in one’s own abilities to overcome the period of detention with all the challenges and to correct behaviors decreases emotional lability, creating a favorable framework for educational and psychotherapeutic interventions, especially since they are both asthenic and easily provoked.

The mediation model showed a decrease in self-efficacy in the inmates in the sample studied because of their coping mechanisms as they aged. At the same time, the years of punishment received and sex have different influences on the activation of coping mechanisms, which are more pronounced in women and when the sentence has fewer years of imprisonment.

The general conclusion of this study is that the implementation of correctional programs and practices in correctional institutions should consider the specificity of personality, coping mechanisms, and self-efficacy that mediate them, as well as the years of detention, sex, and age.

The high level of emotionality and aggression manifested among the inmate in the analyzed sample, associated with the presence of symptoms of stress, anxiety and health problems, underlines the need to implement specialized interventions aimed at restoring emotional balance and developing self-control. Self-efficacy plays a central role in how personality influences the coping mechanisms of prisoners, acting as a mediator in this relationship. Improving the level of self-efficacy can contribute to reducing aggression, anxiety or emotional sensitivity, and can promote prosocial and adaptive behaviors in the penitentiary environment.

Also, intervention measures must be adapted according to identified moderating factors such as gender, length of detention and age, given that stress responses and self-efficacy levels may vary significantly under these conditions.

Substantiating intervention measures taking into account the results of this study contributes to the management of violent behaviors and mental health, both during detention and post-release, thus supporting the reduction of the risk of recidivism and social adaptation.

## Data Availability

The raw data supporting the conclusions of this article will be made available by the authors, without undue reservation.
